# *Mycobacterium iranicum* bacteremia and hemophagocytic lymphohistiocytosis: a case report

**DOI:** 10.1186/s13104-017-2684-8

**Published:** 2017-08-08

**Authors:** Simon Grandjean Lapierre, Alexandre Toro, Michel Drancourt

**Affiliations:** 1 Aix-Marseille Université, URMITE, UMR CNRS 7278, IRD 198, INSERM 1095, IHU-Méditerranée Infection, 19-21 Boulevard Jean Moulin, 13005 Marseille, France; 2 Centre Hospitalier de Martigues, 13500 Martigues, France

**Keywords:** *Mycobacterium iranicum*, Non-tuberculous mycobacteria, Hemophagocytic lymphohistiocytosis

## Abstract

**Background:**

*Mycobacterium iranicum* has recently been recognised as an opportunistic human pathogen. Although infectious conditions represent frequent triggers for hemophagocytic lymphohistiocytosis, non-tuberculous mycobacterial infections are rarely associated with this entity. To this date, *M. iranicum* infection has never been reported in France, has never been associated with hemophagocytic lymphohistiocytosis and has never been found to be multi-resistant on standardized antimicrobial susceptibility testing.

**Case presentation:**

We report a case of a French Caucasian man with secondary hemophagocytic lymphohistiocytosis in the context of *M. iranicum* bacteraemia and Hodgkin’s disease. We review available data concerning *M. iranicum* antimycobacterial susceptibility testing and treatment outcomes. We also review the association between hemophagocytic lymphohistiocytosis and non-tuberculous mycobacterial infections.

**Conclusion:**

Interpretation of *M. iranicum* positive cultures remains a clinical challenge and non-tuberculous mycobacterial infections need to be considered in secondary hemophagocytic lymphohistiocytosis differential diagnosis.

## Background

The American Thoracic Society published clear guidelines of the diagnosis and management guidelines of non-tuberculous mycobacteria (NTM) pulmonary infections [1]. On the other hand, interpretation of NTM positive cultures obtained from non-respiratory specimens is challenging as mycobacterial species and clinical presentations vary greatly. *Mycobacterium iranicum* was recognized as new species in 2013 [2, 3]. Eight human isolates having been retrieved from both healthy and immunocompromised patients were initially reported. These isolates were obtained from specimens including sputum, soft-tissue and cerebrospinal fluid (CSF) in six different countries [2]. Since then, cases of catheter-related peritonitis in a patient with chronic kidney disease, pulmonary infection in a Human immunodeficiency virus (HIV)-positive patient and septic arthritis in a patient with diabetes further confirmed the true pathogenic potential of *M. iranicum* [4–6]. In many cases of *M. iranicum* positive cultures, no treatment was initiated even though *M. iranicum* had been isolated in critical specimens such as CSF.

We report a case of *M. iranicum* bacteraemia with associated hemophagocytic lymphohistiocytosis (HLH) in an immunocompromised patient.

## Case presentation

In July 2016, a 55-year-old French Caucasian man was hospitalized with a 2-week history of fever and weight loss. The patient had no prior medical history of immunosuppression or end-organ disease. Initial clinical evaluation and biological investigations yielded a diagnosis of secondary hemophagocytic lymphohistiocytosis (HLH) fulfilling the HLH-2004 diagnosis criteria [7]. Signs and symptoms included a 40 °C fever, splenomegaly, bi-cytopenia with 8.9 g/dL (N 13.4–16.7 g/dL) haemoglobin and 2.7 g/dL (N 4.0–11.0 g/dL) leucocytes counts, 2.5 g/L (N 0.4–1.5 g/L) hypertriglyceridemia and 8188 μg/L (N 15–100 μg/L) hyperferritinemia. An extended infectious disease work-up was performed to rule out the presence of various HLH associated infections and came back negative for HIV, Epstein–Barr virus, Cytomegalovirus, Hepatitis B virus, Hepatitis C virus and Human Herpes 8 viruses. Routine bacterial cultures were also negative but the investigation revealed *M. iranicum* bloodstream infection and a new diagnosis of Hodgkin’s disease.

*Mycobacterium iranicum* was isolated from a Bact/ALERT™ (bioMérieux, France) mycobacterial blood culture. Following sub-culture on Löwenstein–Jensen and positive pan-mycobacterial internal transcribed sequence confirmation polymerase chain reaction (PCR), matrix-assisted laser desorption-ionisation time-of-flight mass-spectrometry (MALDI-TOF–MS) and *rpoB* gene sequencing-based identification was performed per local identification protocols [8, 9]. Generated proteomic profile did not match in local MALDI-TOF–MS database and *rpoB* gene sequence showed 99.0% similarity with *M. iranicum* strain NLA001001296 (GenBank Accession No. JQ906698.1). Control mycobacterial blood cultures were then obtained and venous catheter was removed and cultivated according to published guidelines [10]. Sputum and urine mycobacterial culture were also obtained. These complementary analyses failed to confirm persistent or disseminated *M. iranicum* bacteremia or catheter-associated bloodstream infection. The patient reported no travel history, animal exposure or other atypical epidemiologic risk factor or environmental exposure. Since no *M. iranicum* isolates were concomitantly handled in our laboratory at the time specimens were obtained, in-laboratory cross contamination is highly improbable. Broth dilution antimicrobial susceptibility testing was performed per published Clinical Laboratory Standard Institute guidelines [11] and showed the isolate to be resistant to clarithromycin, ethambutol, rifabutin and trimethoprim–sulfamethoxazole.

Concomitantly, a diagnosis of stage four Hodgkin’s disease was established on lymph node biopsy and positron emission tomography-scan imaging. Urgent high dose corticosteroids followed by Bleomycin, Etoposide, Adriamycin, Cyclophosphamide, Oncovorin, Procarbazine, Prednisone (BEACOPP) chemotherapy were therefore initiated.

Interpreting whether *M. iranicum* bacteraemia was a trigger for HLH and what was its contribution to the clinical findings was found to be challenging since previous studies established that the isolation of this organism in critical specimens such as CSF may not always require treatment [2]. Since both infectious conditions including disseminated mycobacterial disease and neoplasia including haematological malignancies are recognised triggers of HLH [12] empiric antimycobacterial therapy was highly considered. Patient was followed closely with serial microbiologic investigations. Upon submission of this manuscript, initial HLH biological features had regressed, the patient hadn’t received any anti-mycobacterial treatment, was still alive and undergoing chemotherapy treatment at our institution.

## Discussion

Both phylogenomic and *hsp65*, *rpoB* or 16S rRNA gene targeted phylogenetic analyses have showed *M. iranicum* to be closely related to environmental and rarely pathogenic mycobacterial species [13]. *M. iranicum* was also subsequently isolated from human residential immediate environment [14]. The *M. iranicum* isolate here reported, is the first to be identified in France.

Three to 6 months’ course treatments of aminoglycosides, fluoroquinolones and tetracyclins have been used with success in the management of *M. iranicum* infections in the past [2]. The here reported isolate is the first to present a multi-drug resistance phenotype. *M. iranicum* is believed to have acquired multiple drug resistance genes by horizontal transfer [13]. These include *ermE* which encodes for a multidrug resistance efflux pump in *Escherichia coli* as well as genes coding for metallo-beta-lactamase and macrolide glycosyltransferase. Expression of these resistance mechanisms could partly account for this newly encountered multi-drug resistance profile.

Infections and haematological malignancies are reported as the most common causes of adult HLH. Among the infection induced cases, Epstein–Barr virus is the most frequently reported pathogen [12]. Tuberculosis-associated HLH has frequently been reported especially with disseminated or extra-pulmonary infections. Among these cases, mortality averages 50% and anti-tuberculous or immunomodulatory treatments seem to have no impact on the prognosis [15, 16]. We reviewed every published case of non-tuberculous mycobacterial infections with associated HLH syndrome (Table 1). This rare association seems to occur among patients with underlying immune disorder and organ limited or disseminated mycobacterial infections.

**Table 1:** Non-tuberculous mycobacteria associated hemophagocytic lymphohistiocytosis

	Mycobacterial species	Age	Sex	Infection site	Immune disorder	HLH-2004 criteria [7]	Outcome
Yang et al. [17]	*M. avium* complex	15	F	Pulmonary	Systemic lupus erythematous	Yes	Recovery
Chamsi-Pasha et al. [18]	*M. avium* complex	22	F	Pulmonary	Sickle cell anemia	Yes	Death
Katagiri et al. [19]	*M. abscessus*	58	M	Disseminated	Chronic lymphocytic leukemia	Yes	Recovery
Chou et al. [20]	*M. kansasii*	60	F	Disseminated	None	Yes	Recovery
Javier et al. [21]	*M. xenopi*	N/A	N/A	Disseminated	HIV	Yes	Death
Thomas et al. [22]	*M. lentiflavum*	65	M	Disseminated	Heart transplant	Yes	Death
This case	*M. iranicum*	55	M	Bloodstream	Hodgkin’s lymphoma	Yes	Recovery

## Conclusion

*M* ***ycobacterium*** *iranicum* is a newly recognized species which has been described as a human pathogen in both immunocompromised and previously healthy patients. Although infrequent, mycobacterial infections need to be considered in the context of unexplained hemophagocytic lymphohistiocytosis and *M. iranicum* is amongst the few reported NTM species to be associated with this syndrome. The interpretation of a positive *M. iranicum* culture is challenging, and such is the management of a confirmed *M. iranicum* infection case. Optimal management should be discussed in a multidisciplinary team including both medical microbiologists and infectious diseases specialist as clinical evolution may be unfavourable and antimicrobial resistance may be encountered.

## Abbreviations

BEACOOPP: Bleomycin, Etoposide, Adriamycin, Cyclophosphamide, Oncovorin, Procarbazine, Prednisone
CSF: cerebrospinal fluid
HIV: human immunodeficiency syndrome
HLH: hemophagocytic lymphohistiocytosis
MALDI-TOF: matrix assisted laser desorption/ionization time of flight
NTM: non-tuberculous mycobacteria
PCR: polyerase chain reaction
URMITE: Unité de recherche sur les maladies infectieuses tropicale et émergentes
